# Microglial Activation and Neurological Outcomes in a Murine Model of Cardiac Arrest

**DOI:** 10.1007/s12028-021-01253-w

**Published:** 2021-07-15

**Authors:** Alaa Ousta, Lin Piao, Yong Hu Fang, Adrianna Vera, Thara Nallamothu, Alfredo J. Garcia, Willard W. Sharp

**Affiliations:** Section of Emergency Medicine, Department of Medicine, University of Chicago, 5841 S Maryland Avenue, Chicago, IL 60637 USA

**Keywords:** Ischemia reperfusion injury, Cardiopulmonary resuscitation, Microglia, Brain injuries

## Abstract

**Background:**

Neurological injury following successful resuscitation from sudden cardiac arrest (CA) is common. The pathophysiological basis of this injury remains poorly understood, and treatment options are limited. Microglial activation and neuroinflammation are established contributors to many neuropathologies, such as Alzheimer disease and traumatic brain injury, but their potential role in post-CA injury has only recently been recognized. Here, we hypothesize that microglial activation that occurs following brief asystolic CA is associated with neurological injury and represents a potential therapeutic target.

**Methods:**

Adult C57BL/6 male and female mice were randomly assigned to 12-min, KCl-induced asystolic CA, under anesthesia and ventilation, followed by successful cardiopulmonary resuscitation (*n* = 19) or sham intervention (*n* = 11). Neurological assessments of mice were performed using standardized neurological scoring, video motion tracking, and sensory/motor testing. Mice were killed at 72 h for histological studies; neuronal degeneration was assessed using Fluoro-Jade C staining. Microglial characteristics were assessed by immunohistochemistry using the marker of ionized calcium binding adaptor molecule 1, followed by ImageJ analyses for cell integrity density and skeletal analyses.

**Results:**

Neurological injury in post-cardiopulmonary-resuscitation mice vs. sham mice was evident by poorer neurological scores (difference of 3.626 ± 0.4921, 95% confidence interval 2.618–4.634), sensory and motor functions (worsened by sixfold and sevenfold, respectively, compared with baseline), and locomotion (75% slower with a 76% decrease in total distance traveled). Post-CA brains demonstrated evidence of neurodegeneration and neuroinflammatory microglial activation.

**Conclusions:**

Extensive microglial activation and neurodegeneration in the CA1 region and the dentate gyrus of the hippocampus are evident following brief asystolic CA and are associated with severe neurological injury.

**Supplementary Information:**

The online version contains supplementary material available at 10.1007/s12028-021-01253-w.

## Introduction

Out-of-hospital cardiac arrest (CA) affects as many as 424,000 people annually in the United States and millions worldwide and is associated with high morbidity and mortality [1, 2]. Patients who are successfully resuscitated following cardiopulmonary resuscitation (CPR) often suffer from a sepsis-like syndrome, known as the post-CA syndrome, consisting of cardiovascular and hemodynamic shock, multiorgan failure, neurological injury, and death [3]. Despite improvements in CPR quality and the use of targeted temperature management, neurological injury present in the form of myoclonus and coma is prominent and a major source of morbidity and mortality [4]. The pathophysiological basis of these neurological injuries is poorly understood.

Neuroinflammation is a major contributor to brain injury in the setting of ischemic strokes, neonatal hypoxic encephalopathy, and traumatic brain injury [5–7]. There is a growing body of evidence that progressive neuronal injury continues long after the initial ischemia/reperfusion event and that this injury may be mediated by systemic and localized inflammatory responses in the brain [8, 9]. These inflammatory responses are believed to be mediated by macrophage-like cells in the central nervous system known as microglia. Microglia engage in the secretion of inflammatory cytokines and the phagocytosis of cell debris. When activated, they are typically identified by their increased expression of the cell surface protein called the ionized calcium binding adaptor molecule (Iba1) [10, 11]. However, it is now recognized that activated microglia exhibit distinct morphological changes and that these changes can reflect the degree to which they are activated. The potential reparative vs. pathological role of microglia in brain injury is an area of intense investigation.

The role of microglia in post-CA injury is uncertain. Prior preclinical studies have detected increased Iba1 expression following CA, indicating increased microglial activity [12–20]. These prior studies, however, are lacking because of their (1) lack of morphological analysis of microglia and (2) failure to perform neurological outcome assessments in the setting of presumed microglial activation. In this study, we investigated neurological deficits of mice resuscitated from a brief CA and, using confirmatory methods, determined the extent of microglial activation in this model.

## Methods

### Animals

Adult male and female C57BL/6 mice (aged 79–172 days, weight 17.6–28.7 g) obtained from Charles River Breeding Laboratories (Chicago, IL) were used in the experiments. Animals were housed in the University of Chicago Animal Research Resources Center. A temperature of 22–24 °C was maintained on a 12:12-h light/dark cycle, and food (standard mouse chow) and water were provided ad libitum.

### Animal Model of CA

**Fig. 1:**
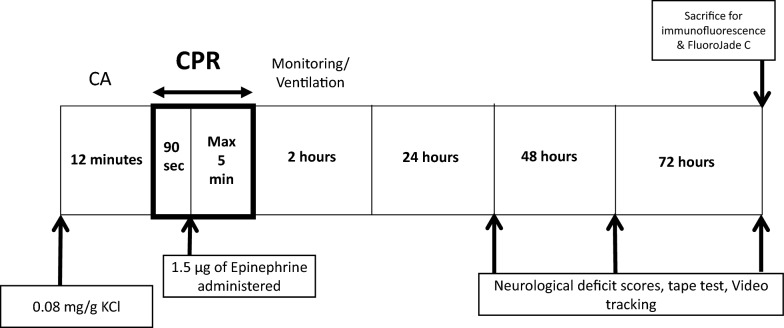
CA model and experimental time line. CA cardiac arrest, CPR cardiopulmonary resuscitation, KCl potassium chloride

We used a recently published modified version of a murine model of asystolic CA because of its reproducibility and precise timing [21] (Fig. 1). All procedures were approved by the Institutional Animal Care and Use Committee of the University of Chicago, in accordance with the National Institutes of Health guidelines. Mice were anesthetized with 3% isoflurane, and intubated and vascular access was acquired. Ventilatory parameters include a tidal volume of 12.5 ml/g, a positive end-expiratory pressure of 2 cm H_2_O, and a rate of 110 breaths per minute. Temperature and electrocardiography tracings were recorded continuously on a PowerLab data acquisition module (AD Instruments, Colorado Springs, CO). A 0.4-mm OD heparinized micropolyethylene cannula (BioTime Inc, Berkeley, CA) was placed in the left jugular vein for fluid administration and right carotid artery for aortic systolic pressure measurement. Mice undergoing surgery were randomly assigned into either a CA group or sham group. Asystolic CA was induced with a single bolus of KCl (0.8 mg/g body weight) into the internal jugular vein, and ventilation was suspended. Following 12 min of CA, CPR was performed. After 90 s of CPR, 1.5 μg of epinephrine was injected followed by a saline flush. CPR was terminated when return of spontaneous circulation (ROSC), defined by a mean arterial pressure greater than 40 mm Hg lasting longer than 5 min, was achieved. CPR was terminated if ROSC was not achieved after 5 min. The quality of CPR was retrospectively evaluated for each animal by reviewing CPR rates and arterial pressures. Only animals that achieved ROSC were included in the study. Sham animals underwent similar operations as the CA animals but without the induction of CA. Control (naïve) animals did not undergo any surgery. Animals were then returned to the animal facility. All surviving animals were killed at 72 h, per our previously established animal protocol, and to allow for tissue analysis.

### Neurological Function Score

General neurological assessment of mice was performed using a modified rodent neurological scoring system previously published [21–24]. The score has a list of six domains: paw pinch, righting reflex, breathing, spontaneous movement, motor global, and motor focal. Each domain has a score ranging from 0 (no response) to 2 (normal response), giving a total score of 12. The scores for each of the six domains were determined in a blinded fashion and summed to calculate the neurological score. Baseline neurological scores were taken before the intervention. Then the scores were taken at 24, 48, and 72 h after intervention. Mice found dead at either of the time points were excluded for the time point as well as for future measurements.

### Tape Test

To assess sensory function and motor strength, a modified version of a previously published tape test protocol was used [25]. A 5 mm × 5 mm adhesive tape is used and modified in a way that avoids frequent falls and makes it hard to remove, especially with a normal, uninjured mouse. We placed the adhesive on one of the forepaws of the mouse, and timed measurements were made in a chamber that it had been familiarized with. Two durations of time were measured. Time to attempt removal of the tape was the time needed by the mouse to touch the tape. This reflected the time needed by the mouse to sense the tape. The second duration measured was the time to successful removal of the tape, which reflected the motor function of the mouse. A cutoff of 180 s was determined based on previously published studies [26]. Baseline measurements were taken before the intervention. After the intervention, measurements were taken in a blinded fashion. Mice that were found dead at either of the time points were excluded for the time point as well as for future measurements.

### Video-Tracking Analysis

Locomotion of mice was assessed using the CineLAB Video Tracking System (Plexon Inc, Dallas, TX). At 24, 48, and 72 h after the intervention (CA or sham operations), the mice were taken to the quiet setup with minimal surrounding stimuli for a 2-min video assessment. During video-tracking, mice were left to travel around freely. The videos were then analyzed to measure the average speed in meters per second and the total length traveled in meters.

### Fluorescence Staining

Brain tissue was prepared for staining as described in a previously published protocol [27]. At 72 h after ROSC/intervention, anesthetized CA mice, control mice (i.e., naïve, un-intervened mice), and sham mice underwent transcardial perfusion with 50 ml of 1% phosphate-buffered saline (PBS) followed by 50 ml of 4% PFA. Brain tissue was analyzed at 72 h. This time point used was based on our prior study that noted increased brain glucose use 72 h post CA indicated possible inflammation [23]. Brains were resected, kept in 4% PFA overnight at 4 °C, and then switched to 30% sucrose (in PBS) solution for 2 days. Subsequently, the brains were frozen in optimal cutting temperature compound and kept in an − 80 °C environment. Blocks containing a single hemisphere from each animal were coronally sectioned at a thickness of 40 μm, and every 12th section was sampled. Each animal in the study had at least three usable sections through the septal region of wither, the dentate gyrus, or the CA1 area.

Tissue slices were simultaneously subjected to Fluoro-Jade C staining using the Fluoro-Jade C Ready-to-Dilute Staining Kit (Biosensis, Temecula, CA) and following the manufacturer’s instructions. Slides were visualized under the confocal microscope, and Fluoro-Jade C-positive neurons were counted using the Fiji software from ImageJ (ImageJ 1.5; National Institutes of Health, Bethesda, MD).

Immunohistochemistry was also performed in separate experiments, as described in previous studies [27]. Slices were randomly assigned in a standardized fashion for all groups, and staining was performed simultaneously. After three 5-min PBS washes, antigen retrieval with sodium citrate, and blocking in 20% donkey serum, we incubated the slices overnight at 4 °C with the primary antibody, anti-Iba1 (1:1000; catalog #019-1974, CAM6570; FujiFilm). The next day, slices were incubated with the fluorescent rhodamine-conjugated secondary antibody at room temperature for 2 h. After three 20-min PBS washes, we incubated the tissues in 4′6-diamidino-2-phenylindole for 2 min. After mounting, slides were visualized under the confocal microscope. Images were analyzed using ImageJ for fluorescence intensity. Therefore, we used fluorescence intensity to quantify how much protein is expressed, i.e., the specific antibodies used for staining fluoresce when targeting Iba1. Iba1 expression is one way of evaluating microglial activity.

### Microglial Morphological Analysis

Next, to determine the extent of microglial activation, objective morphological analysis of Iba1 cells from the acquired images was performed. Microglial activity ranges from quiescent to active phagocytosis, and these activities are reflected in their morphology. Quiescent microglia are characterized by a high degree of ramification (branches) with long lengths, whereas active phagocytosing microglia are characterized by a low degree of ramification with short branches. Images were analyzed using a previously published protocol [28]. Briefly, images of microglial cells were converted to binary images and then skeletonized using ImageJ software. Skeletal analysis was used to analyze the degree of ramification (number of branches) of each microglial cell as well as the length of each ramification (branch).

### Statistical Analyses

All data are presented as mean ± standard error of mean and were analyzed using GraphPad Prism 7 (GraphPad Software, San Diego, CA). Sample sizes for our experiments were estimated through an a priori power analysis by our institution’s biostatistician using PASS Software. The mixed-model method was used to compare groups in the neurological function scores and the sensory and motor tape tests, accounting for time. The mixed-model method is a modified two-way analysis of variance (ANOVA) method that accounts for subsequently lost values. This method is considered the most suitable given that dead animals’ scores were subsequently removed beyond the point when they were last found alive. The one-way ANOVA was used to compare groups in the video-tracking analyses of total distance traveled and average speed of animals. The one-way ANOVA was also used for histopathological analyses, using Dunnett’s multiple comparison tests for post hoc analysis when comparing each of the CA and sham groups with the control group. A log-rank test was used for survival analysis. *p* values less than 0.05 were considered statistically significant.

## Results

### General Characteristics, Survival Rates, and Overall Neurological Scores

**Fig. 2:**
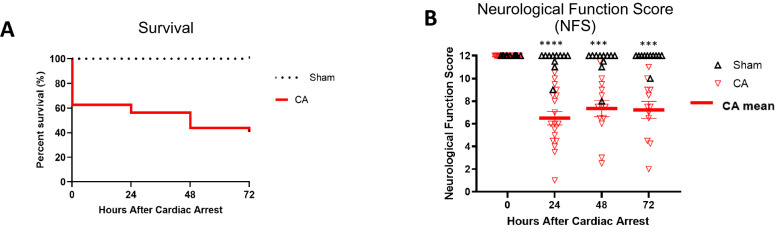
Survival rates and NFS. **a** Post-CA survival curve: log-rank test (*p* = 0.0003). **b** Post-CA neurological function scores of animals over time (0 = poor, 12 = normal). *n* (sham) = 11 at 0, 24, 48, and 72 h. *n* (CA) = 19, 18, 13, and 12 at 0, 24, 48, and 72 h, respectively. Type III tests of fixed effects for time × intervention showed a significant difference between predicted means (*p* < 0.0001). Data are expressed as mean ± SEM. CA cardiac arrest, NFS neurological function score, SEM standard error of mean

A total of 42 mice were used and randomly assigned into either the sham or CA group; 11 of those mice were used for sham surgeries. Among the 31 mice that underwent CA, 61% (*n* = 19) attained ROSC. Only post-CA mice that attained ROSC were included in our experiments (Supplemental Table). Survival rates at 24, 48, and 72 h were 55% (17 mice), 43% (13 mice), and 39% (12 mice), respectively (log-rank test *p* = 0.0003; Fig. 2a), similar to our previous studies [21, 23, 29]. General characteristics of the mice, including weight, age, and CPR quality, are described (Table 1). Baseline characteristics were all taken only before any intervention. Weight by age and sex was used to ensure those baseline characteristics were similar in our experimental groups and was not used to evaluate post-CA characteristics. Among the sham mice, six were female and five were male (54.5% and 45.5%, respectively). Among the CA mice with ROSC right after the intervention, ten were female and nine were male (52.6% and 47.4%, respectively). At 72 h, among those CA mice found alive, ten were female, and two were male. Although at 72 h post CA we did see increased survival in females vs. males, these results were not statistically significant (*p* = 0.1204; Supplemental Fig.ure1). There was also no significant difference in survival rates between mice that were less than 90 days old and mice that were older than 90 days (*p* = 0.2422; Supplemental Figure ). Among the CA survivors, weight differences were not significant between males and females (*p* = 0.0875) or between mice less than 90 days of age and mice more than 90 days of age (*p* = 0.6621).

**Table 1:** Baseline characteristics of successfully resuscitated mice

	Sham (*n* = 11)	CA (*n* = 19)	*P* value
Sex, *n*			
Female	6	10	–
Male	5	9	–
Weight (g)	23.34 ± 0.82	23.84 ± 0.62	0.63
Age (d)	108.6 ± 9.02	109.1 ± 6.38	0.97
HR (bpm)	545 ± 14.04	518 ± 13.92	0.21
ASP (mm Hg)	132.3 ± 3.69	133.7 ± 3.35	0.78
ADP (mm Hg)	93.6 ± 3.02	98.36 ± 1.82	0.16
CPR rate (bpm)	NA	351.2 ± 2.73	–
CPR–ASP (mm Hg)	NA	67.94 ± 1.46	–
CPR–ADP (mm Hg)	NA	25.69 ± 1.39	–
Time to ROSC	NA	134.9 ± 8.4	–

Results presented as mean ± SEM

ADP, aortic diastolic pressure; ASP, aortic systolic pressure; CA, cardiac arrest; CPR, cardiopulmonary resuscitation; bpm, beats per minute; HR, heart rate; NA, xxx; ROSC, return of spontaneous circulation; SEM, standard error of mean

The fixed effects of CA accounting for time showed significantly worse neurological scores, with a predicted mean of 8.101, compared with those of sham mice, with a predicted mean of 11.73 (*p* < 0.0001, 95% confidence interval [CI] 2.618–4.634) (Fig. 2b). In particular, global motor function was affected with a difference between predicted means of 0.8289, paw pinch with a difference between predicted means of 0.6517, and spontaneous movements with a difference between predicted means of 0.6023.

### Sensory Deficits, Motor Deficits, and Locomotion

**Fig. 3:**
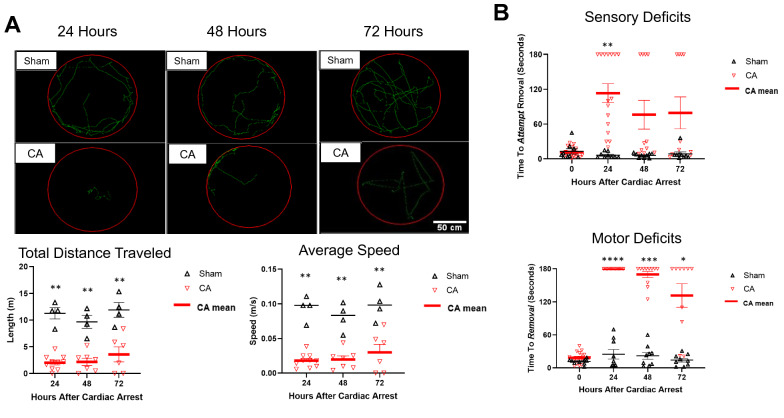
Locomotive and tape test results. **a** Locomotive deficits: mapped tracks at 24, 48, and 72 h for each of the sham and CA groups. Post-CA average speed of animals and total distance traveled over time. *n* (sham) = 4 at 24, 48, and 72 h. *n* (CA) = 10, 7, and 6 at 24, 48, and 72 h, respectively. A paired *t* test showed significant differences at each time point (*p* = 0.0038 for average speed and *p* = 0.0036 for total distance traveled). **b** Tape test results: post-CA sensory deficits reflected through time to attempt removal of the tape, and motor deficits reflected through time to successful removal of tape. Data are expressed as mean ± SEM. Type III tests of fixed effects for time × intervention showed significant differences between predicted means (*p* = 0.0004 for sensory deficits and *p* < 0.0001 for motor deficits). CA cardiac arrest, SEM standard error of mean

Sensory and motor deficits of sham and CA mice were measured using the tape test (Fig. 3b). The fixed effects of CA accounting for time showed a significant increase in the needed time to attempt tape removal (sensory deficit), with a predicted mean of 74.37 s, compared with the time needed among sham mice, which had a predicted mean of 8.583 s (*p* = 0.0004, 95% CI − 96.21 to − 35.37). Post-CA mice also needed more time to successfully remove the tape (motor deficits), with a predicted mean of 125.3 s, compared with sham mice, which had a predicted mean of 18.08 s (*p* < 0.0001, 95% CI − 120.7 to − 93.61). Video-tracking experiments further demonstrated that post-CA mice (*n* = 10, 7, and 6 at 24, 48, and 72 h, respectively) traveled a much shorter distance compared with sham mice (*n* = 4 at 24, 48, and 72 h), with a mean of differences of 8.361 m (*p* = 0.0036; Fig. 3a). Post-CA mice were also much slower than sham mice, with a mean of differences of 0.06890 m/s (*p* = 0.0038; Fig. 3a).

### Neurodegeneration

**Fig. 4:**
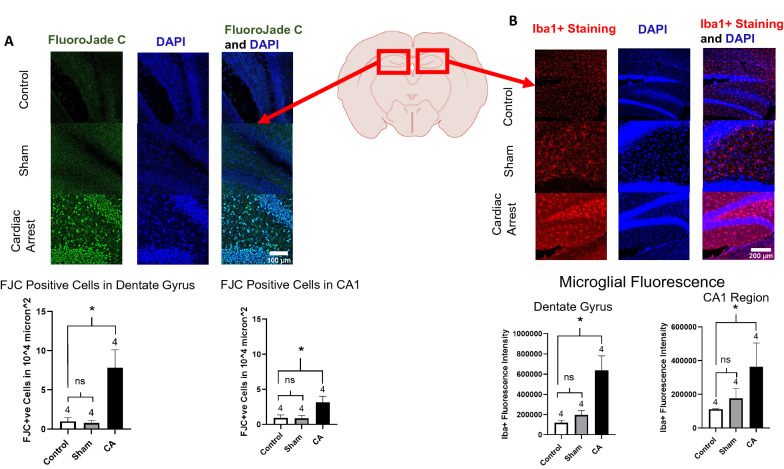
Neurodegeneration and neuroinflammation. **a** Neurodegeneration: representative microphotographs of Fluoro-Jade C-stained brain slices (dentate gyrus of hippocampi, × 10 magnification) in control and CA groups at 72 h after intervention. Scale bar = 100 μm. Fluoro-Jade C staining showed a significant increase in neurodegeneration in CA mice brain tissue (7.8 times more dead cells within the dentate of CA hippocampi and 3.3 times more within the CA1 area, compared with control hippocampi per 10^4^ μm^2^ within the polymorphic layer of the dentate gyrus). Data are expressed as mean ± SEM. One-way ANOVA showed significant differences with *p* < 0.05. **b** Neuroinflammation: representative microphotographs of Iba1 immunofluorescence-stained microglia in control and CA brain slices (dentate gyrus of hippocampi  × 10 magnification) at 72 h after intervention. Scale bar = 200 μm. Immunofluorescent staining showed a significant increase in integrity density in CA mice brain tissue (both dentate gyrus and CA1 areas), reflecting a higher expression of Iba1 and, hence, different microglial morphology. Data are expressed as mean ± SEM. One-way ANOVA showed significant differences with *p* < 0.05. ANOVA analysis of variance, CA cardiac arrest, DAPI, xxx, FCJ, xxx, Iba1 ionized calcium binding adaptor molecule 1, ns, xxx, SEM standard error of mean

Neurodegeneration was assessed by Fluoro-Jade C staining of brain slices from post-CA mice, sham mice, and control (naïve) mice (*n* = 4 mice in each group, 3–4 tissues per mouse; Fig. 4a). We analyzed the polymorphic granular area of the dentate gyrus and CA1 area of the hippocampi, counted the cells superimposed with both DAPI and Fluoro-Jade C, and normalized the counts to surface area (10^4^ μm^2^). CA brain tissues exhibited a significant increase in neurodegeneration compared with control brain tissues in the dentate gyrus (*p* = 0.0087) and the CA1 area (*p* = 0.0355), using Dunnett’s multiple comparisons to control the one-way ANOVA, and there was no significant difference between control and sham tissue in either area (*p* = 0.99 in the dentate and *p* = 0.99 in the CA1 area).

**Fig. 5:**
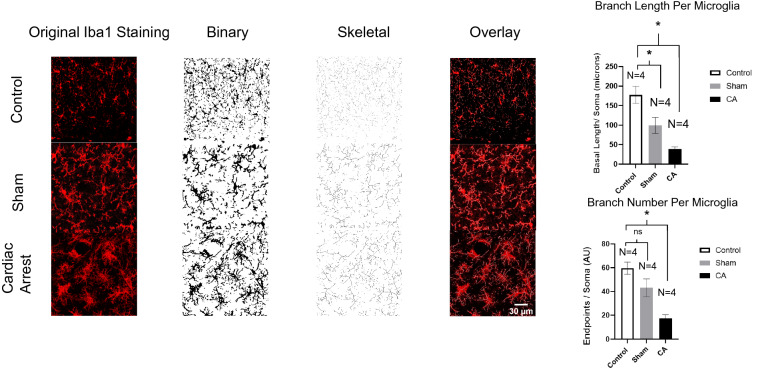
Describing microglial morphologies. Representative microphotographs of Iba1 immunofluorescence-stained microglia in control and CA brain slices (hippocampi, × 20 magnification) at 72 h after intervention. The images were modified using the ImageJ software (to binary, skeletal, and overlay images) for skeletal analysis. Scale bar = 50 μm, *n* = 4 per group. The degree of ramification was less complex in CA microglia compared with control microglia in terms of branch length and branch number per microglia. Sham microglia showed some degree of activation in terms of branch length but not number of branches. Data are expressed as mean ± SEM. One-way ANOVA showed significant differences with *p* < 0.05. ANOVA, analysis of variance, CA, cardiac arrest; N, number of animals.

### Inflammation and Microglial Characteristics

Neuroinflammation was assessed by Iba1 staining and morphological analysis of Iba1 cells in the hippocampus (Fig. 4b). Iba1 is a widely recognized marker, mostly expressed on activated microglia and, to a lesser extent, on macrophages [30]. CA brain tissues expressed an Iba1 fluorescence intensity significantly more robust than what was expressed in the control or sham brain tissues (*n* = 4 mice in each group, 3–4 tissues per mouse), demonstrating significant microglial activation (*p* = 0.0051 in dentate areas and *p* = 0.0297 in the CA1 areas). There was no significant difference between the control and sham mice in the dentate (*p* = 0.78) or CA1 areas (*p* = 0.3956).

Although Iba1 expression is indicative of overall microglial activity, assessing microglial morphology has been proven to be a more reliable and sensitive method to determine the degree of microglial inflammatory activation and, hence, inflammation in the area of interest. Activated microglia are characterized by their assumption of an ameboid and less complex shape. Two major characteristics help quantify their degree of complexity: the degree of ramification and the length of the branches. Recently, new protocols for scanning all the microglia in a single photographic field at once and objectively assessing the morphology of each individual microglial cell through skeletal analysis have become available. Using this approach, we were able to study the in-depth features of microglia [28, 31] in the hippocampus from the control (naïve, nonoperated), sham (operation but no CA), and post-CA mice.

Microglia in the hippocampus of post-CA mice demonstrated shorter process lengths per soma by almost 78% (*p* = 0.0013) and fewer process end points per soma by 70% compared with microglia in naïve brain tissue (*p* = 0.0018).

Comparing microglia in sham with that in naïve brain tissue, it seems that the process lengths were significantly shorter (*p* = 0.02) but not significantly less (*p* = 0.12). The described characteristics demonstrate that microglia in post-CA brains are less complex than control microglia and are consistent with inflammatory activation (Fig. 5).

## Discussion

In this study, we made three important discoveries. First, we definitively confirmed central nervous system microglial activation following brief CA using immunofluorescent Iba1 staining and microglial morphometric analysis. Second, we determined that these inflammatory changes occurred in the context of prominent neurological injury. Motor injuries were particularly more prevalent than sensory deficits in the post-CA animals. Third, we discovered that neuronal degeneration in the hippocampus was not limited to the CA1 region but was also prevalent in the dentate gyrus.

### Neuronal Microglial Activation Following CA

Consistent with previous studies of neurological injury in CA, we found evidence of increased microglial activation, as assessed through Iba1 staining [12–20]. Microglial staining was prominent in both the CA1 region and dentate gyrus of the hippocampus 72 h following CA and was associated with neurodegeneration. Increased Iba1 microglial staining has been noted in many neurological pathologies and has been noted to persist as long as 90 days post CA [32–34]. Our results are consistent with these prior studies demonstrating Iba1 microglial staining.

Microglia are highly dynamic cells with different degrees of activation. Microglial activation can range from low, or quiescent, surveillant activity in the brain to a more highly active, macrophage-like activity, representing active phagocytosis of cellular debris. These different ranges of microglial activity are more accurately reflected by changes in microglial morphology than by mere increases in Iba1 expression. Highly activated microglia are deramified with fewer branches than in their less active states [35, 36]. These morphological changes are now easily assessed using recently established morphological analysis techniques [28]. In this study, for the first time, we demonstrate that post-CA injury is associated not only with increased Iba1 expression but also with large changes in microglial morphology reflective of their highly activated state.

### Neurological Deficits Following CA

In previous studies, we and others have demonstrated that CA induces neurological injury, the severity of which can depend on the length of the CA [8, 12, 21]. In this study, we used a 12-min asystolic CA. This is the longest length of CA in our studies that results in the most severe neurological injury with enough surviving animals to practically study outcomes [21]. To date, most studies on microglial activation following CA fail to report any neurological outcomes in the context of their findings. Thus, it is uncertain whether microglial activation and neuronal degeneration in these studies were associated with actual neurological injury.

Using a previously published standardized neurological scoring tool, we determined the general extent of injury in our animal studies [21, 23, 29]. We discovered that post-CA mice exhibiting microglial activation, indeed, had neurological injury that was severe and persistent. To further gauge the severity of neurological injury, we used video-tracking analysis. This approach has been previously used in stroke outcome analysis but not in post-CA outcome studies [37]. Total distance and speed of post-CA animals were severely reduced by 76% and 75%, respectively, compared with total distance and speed sham-operated animals. This reflects severe neurological injury. Finally, we investigated the degree of motor and sensory injury in post-CA animals using a standardized tape removal test [26]. We discovered that there was a sixfold increase in the time to attempt to remove the tape, reflecting impairment in sensory detection. We also discovered a sevenfold increase in the time to remove the tape, demonstrating impaired motor activity.

Our approach of using neurological scoring, video surveillance tracking, and specific measurements of sensory and motor functions demonstrates that severe neurological injury and generalized encephalopathy occur following 12-min asystolic CA and that these injuries are associated with a high degree of microglial activation and neuroinflammation. Although the neurological assessments used in this study were not specific to any one area of the brain, they do evaluate the integration of the cortex with other areas of the brain, including the basal ganglia, the cerebellum, or the hippocampus [38, 39]. Unfortunately, the resulting injury following 12-min CA in this model was too severe to allow for more extensive neurocognitive testing or for the study of long-term outcomes. Future studies using neurocognitive maze testing to better assess long-term learning, memory, and spatial navigation outcomes, using a shorter length of CA to produce less severe outcomes, are needed.

### Neurodegeneration Following CA

Finally, we looked for evidence of neurodegeneration in the setting of microglial activation and neurological injury following CA by examining the hippocampus, which is a brain region well recognized for vulnerability to ischemia [2, 13]. Similar to prior studies, we discovered evidence of neuronal cell death in the hippocampal CA1 region [12, 13]. More significantly, we further discovered that this injury was not limited to the CA1 region but was also evident in the dentate gyrus of the hippocampus. These finding are significant for two reasons. First, the dentate gyrus is a unique area of the mammalian brain in which adult neurogenesis occurs. Adult neurogenesis can be considered a natural form of self-renewal and may facilitate neuronal repopulation following ischemic injury in the CA1 region [40]. The extent of injury found in our model suggests the low likelihood of postinjury neurogenesis and the irrevocability of the injury severity. Second, the dentate gyrus is the first network of the hippocampal circuit receiving cortical input that is ultimately relayed to the CA1 area. The dentate is, thus, critical for integrating cortical-hippocampal signals. The injury discovered in our study suggests further explanation for the encephalopathy exhibited by the post-CA animals.

### Limitations and Future Studies

There are several important limitations to our study. First, the study was performed using an asystolic model of CA. Although the lengths of arrest and ischemia are key determinants of injury, we cannot exclude whether our findings are applicable to other forms of CA (asphyxial/ventricular fibrillation). Second, our findings were in mice. Although mice are commonly used to study neurological disorders, findings in these animals may not directly reflect those in animals with more complex neurological function, such as human patients. Third, our findings were limited to a relatively short duration (72 h) post CA. This was due to the severity of injury produced by the long duration of CA. Future studies investigating outcomes using shorter cardiac durations and longer-term outcomes are needed. Fourth, as with all preclinical animal studies, the number of subjects was limited. To ensure our studies were powered adequately, we consulted with a biostatistician to guide our studies. The model that was employed (mice) also allowed a relatively large number of subjects to be evaluated, compared with other models (porcine). However, caution must be used in interpreting our results. Fifth, a statistically nonsignificant trend for improved survival in female mice vs. male mice was noted in a subgroup analysis following our study. However, our study was underpowered to determine the influence of sex on post-CA outcomes, and future studies are needed to study the potential influence on outcomes.

## Conclusions

Severe neurological injury and encephalopathy occur following CA that is associated with extensive microglial activation and neurodegeneration in the CA1 region and the dentate gyrus of the hippocampus. Future studies investigating this inflammatory response and its potential therapeutic target to improve post-CA outcomes are needed.

## Supplementary Information

Below is the link to the electronic supplementary material.

Supplementary file1 (PPTX 822 KB)

Supplementary file2 (PPTX 813 KB)

Supplementary file3 (DOCX 14 KB)
