# Supplementary material for: Microglial Activation and Neurological Outcomes in a Murine Model of Cardiac Arrest
Source: Neurocrit Care. 2021 Jul 15;36(1):61–70. doi: 10.1007/s12028-021-01253-w (PMC8813848; doi:10.1007/s12028-021-01253-w)
Supplement: Supplementary file 2 — Supplementary file2 (PPTX 813 KB) [file 12028_2021_1253_MOESM2_ESM.pptx]

## Slide 1
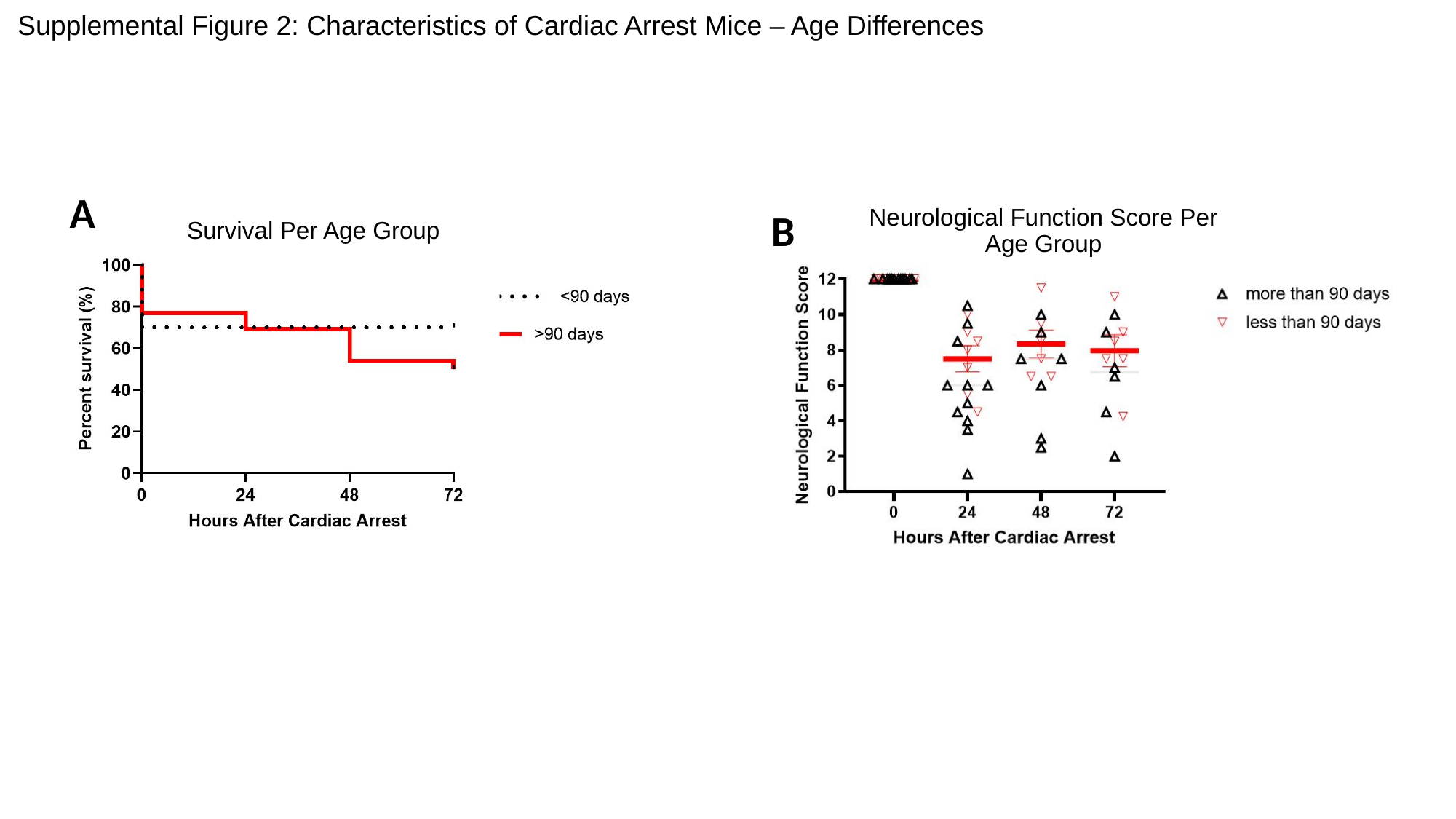

Supplemental Figure 2: Characteristics of Cardiac Arrest Mice – Age Differences
A
B
Survival Per Age Group
Neurological Function Score Per Age Group
