# Supplementary material for: Microglial Activation and Neurological Outcomes in a Murine Model of Cardiac Arrest
Source: Neurocrit Care. 2021 Jul 15;36(1):61–70. doi: 10.1007/s12028-021-01253-w (PMC8813848; doi:10.1007/s12028-021-01253-w)
Supplement: Supplementary file 3 — Supplementary file3 (DOCX 14 KB) [file 12028_2021_1253_MOESM3_ESM.docx]

Supplemental Table: Number of Survivors at Each Experimental Stage

| Experimental Stage | CA/CPR | Sham |
| --- | --- | --- |
| Initial number | 31 | 11 |
| ROSC | 19 | 11 |
| 24 hours | 17 | 11 |
| 48 hours | 13 | 11 |
| 72 hours | 12 | 11 |
